# Sensitivity to a Break in Interaural Correlation in Frequency-Gliding Noises

**DOI:** 10.3389/fpsyg.2021.692785

**Published:** 2021-06-17

**Authors:** Langchen Fan, Lingzhi Kong, Liang Li, Tianshu Qu

**Affiliations:** ^1^Beijing Key Laboratory of Behavior and Mental Health, School of Psychological and Cognitive Sciences, Peking University, Beijing, China; ^2^Key Laboratory on Machine Perception (Ministry of Education), Department of Machine Intelligence, Peking University, Beijing, China; ^3^Language Pathology and Brain Science MEG Lab, School of Communication Sciences, Beijing Language and Culture University, Beijing, China

**Keywords:** auditory system, binaural hearing, center frequency, interaural correlation, frequency gliding, interaural delay

## Abstract

This study was to investigate whether human listeners are able to detect a binaurally uncorrelated arbitrary-noise fragment embedded in binaurally identical arbitrary-noise markers [a break in correlation, break in interaural correlation (BIAC)] in either frequency-constant (frequency-steady) or frequency-varied (unidirectionally frequency gliding) noise. Ten participants with normal hearing were tested in Experiment 1 for up-gliding, down-gliding, and frequency-steady noises. Twenty-one participants with normal hearing were tested in Experiment 2a for both up-gliding and frequency-steady noises. Another nineteen participants with normal hearing were tested in Experiment 2b for both down-gliding and frequency-steady noises. Listeners were able to detect a BIAC in the frequency-steady noise (center frequency = 400 Hz) and two types of frequency-gliding noises (center frequency: between 100 and 1,600 Hz). The duration threshold for detecting the BIAC in frequency-gliding noises was significantly longer than that in the frequency-steady noise (Experiment 1), and the longest interaural delay at which a duration-fixed BIAC (200 ms) in frequency-gliding noises could be detected was significantly shorter than that in the frequency-steady noise (Experiment 2). Although human listeners can detect a BIAC in frequency-gliding noises, their sensitivity to a BIAC in frequency-gliding noises is much lower than that in frequency-steady noise.

## Introduction

The auditory system usually implicates functions of two ears, integrating the sound information from both ears. The binaural hearing has been recognized as a critical function of the central auditory system, offering substantial advantages in localizing sounds, dealing with reflections, and improving speech recognition in adverse environments (Kohlrausch et al., 2013).

Interaural coherence (the degree of similarity of the sound waveforms at the two ears) can be physically measured as the maximum value of the cross correlation between the sound wave at the left ear and the sound wave at the right ear when one of the two sounds has been time shifted (within limits, e.g., ±1 or ±2 ms) to maximize the correlation (Grantham, 1995; Aaronson and Hartmann, 2010), which is called “interaural correlation.” If the sound wave at the left ear is an identical copy of the wave at the right ear, the interaural correlation is one. In contrast, if the sound waves at the left and right ears are independently generated, the interaural correlation is near to zero. The interaural correlation can be represented at both the neurophysiological level (Wang et al., 2018) and the perceptual level (Blauert and Lindemann, 1986). When sounds, i.e., arbitrary noises, arrive at the two ears simultaneously, identical sounds (interaural correlation = 1) at the two ears are perceptually fused into a single image at the center area of the head, while binaurally independent sounds are perceived as two separated sound images at each ear (Blauert and Lindemann, 1986).

Several previous studies have shown that human listeners are able to discriminate changes in the interaural correlation across two binaural noises. Particularly, the discrimination was extremely sensitive to a slight drop in the interaural correlation from binaurally identical noise (with an interaural correlation of one; Pollack and Trittipoe, 1959; Gabriel and Colburn, 1981; Akeroyd and Summerfield, 1999; Culling et al., 2001; Boehnke et al., 2002; Chait et al., 2005). Furthermore, the sensitivity to the dynamic change in interaural correlation has been investigated using a binaural analog of the gap-detection paradigm by placing a binaurally uncorrelated fragment, i.e., a break in interaural correlation (BIAC; a pair of binaurally independent noises), in the temporal center of two bursts of binaurally identical noise (markers: Akeroyd and Summerfield, 1999; Boehnke et al., 2002). Introducing a BIAC does not alter the energy or spectrum of the arbitrary noise but modifies the auditory images, including the perceptual compactness/diffuseness of the noise image (Blauert and Lindemann, 1986; Edmonds and Culling, 2009). The duration threshold (the minimum duration required to detect a BIAC) is measured to determine the sensitivity to a dynamic change in interaural correlation. Previous studies have proved that human listeners are sensitive to a BIAC in either broad-band or narrow-band noise whose spectral information does not vary with time monaurally (Akeroyd and Summerfield, 1999; Boehnke et al., 2002).

Moreover, understanding of the interaural correlation processing is incomplete without considering the impact of the interaural delay. As the interaural delay increases from zero, the perceptually fused single auditory image of binaurally identical noise initially moves toward the leading ear, then becoming increasingly diffuse and eventually indistinguishable from the sound image of the binaurally independent noise (Blodgett et al., 1956). Our previous studies have shown that the sensitivity to a BIAC decreased dramatically as the interaural delay increased from zero to several milliseconds, and the maximum interaural delay, at which a BIAC can be detected (the delay threshold), has been used to determine the impact of the time delay between the sounds at the two ears on the sensitivity to a change in interaural correlation (Huang et al., 2008, 2009a,b, 2019; Li et al., 2009, 2013; Kong et al., 2012, 2015; Qu et al., 2013).

Ecologically, communication sounds with time-varying spectra are common for humans and other species. For example, the frequency modulation is a fundamentally acoustic component in human speech, critical to the discrimination of vowels (Jenkins et al., 1983), the recognition of Mandarin tones (Kong and Zeng, 2004), and the speech recognition in noise (Zeng et al., 2005).

Moreover, it has been shown that the auditory system is sensitive to the binaural cues even in frequency-gliding tone (frequency range: 3–8 kHz; Hsieh and Saberi, 2009). To our knowledge, however, the issue of the sensitivity to changes in interaural correlation in frequency-gliding sound has not been reported.

Previous binaural models share a fundamental notion that binaural performance, e.g., interaural correlation processing, is based on frequency-band-by-frequency-band comparisons of bandpass-filtered signals at two ears (Stern and Trahiotis, 1995; Ungan et al., 2019). The frequency selectivity of binaural processing is not necessarily poorer than that for monaural processing (Verhey and van de Par, 2018), since it has been shown that the auditory system is capable of integrating binaural information across different frequency channels (Jain et al., 1991; Hsieh and Saberi, 2009). Thus, we hypothesized that human listeners can hear a dynamic change in interaural correlation in noises with center frequency varying unidirectionally when both the spectral and temporal integrations are involved.

## Materials and Methods

### Participants

All participants were young adult university students at the Peking University. They had pure-tone thresholds no higher than 25 dB HL between 0.125 and 8 kHz, and the threshold difference between the two ears at each testing frequency was less than 15 dB HL. They gave written informed consent and were paid a modest stipend for their participation. All the experimental procedures were approved by the Committee for Protecting Human and Animal Subjects in the School of Psychological and Cognitive Sciences at Peking University.

Ten participants (eight females, 18–26 years old, mean age = 20.5 years) took part in Experiment 1. Twenty-one different participants (15 females, 17–24 years old, mean age = 19.1 years) were tested in Experiment 2a. Another group of the participants (13 females, 18–27 years old, mean age = 20.7 years), who did not participate in Experiment 1 and Experiment 2a, were tested in Experiment 2b.

### Apparatus and Stimuli

The participant was seated in a chair at the center of a sound-attenuated chamber (EMI Shielded Audiometric Examination Acoustic Suite). Frequency-steady and frequency-gliding noises (sampling rate = 16 kHz; duration = 2,000 ms; rise/decay time = 50 ms) were synthesized using MATLAB (the MathWorks Inc., Natick, MA, United States).

**Figure 1:**
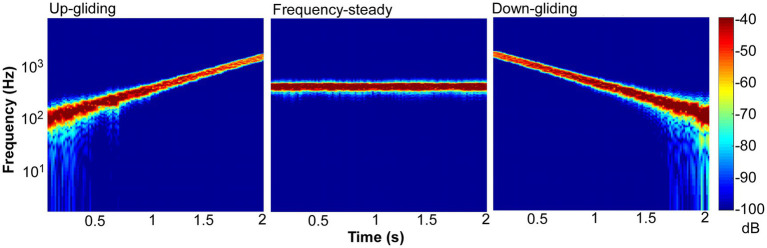
Spectrograms of up-gliding, frequency-steady, and down-gliding noises.

To produce frequency-steady noise, Gaussian wideband noise (0–8 kHz) was generated and bandpass filtered (the 400-Hz geometric center frequency with a bandwidth of 1.585 octave). To produce frequency-gliding noises, the wideband noises were cut into temporal frames using a Hanning window. The frame length was 62.5 ms (duration of on/off ramps = 31.25 ms) and the frameshift time was 15.6 ms (with an overlap between successive frames). The energy of each of the frames was set to a fixed value. These wide-band noise frames were filtered into narrow-band noise frames by a 1.585-octave wide bandpass filter in the frequency domain, and the frequency components outside the passband were set to zero. Each narrow-band frame had a center frequency in the range from 100 to 1,600 Hz (log spaced). The sequence of the center frequencies was from 100 to 1,600 Hz or from 1,600 to 100 Hz for the up-gliding and down-gliding noises, respectively. Note that all the frames were concatenated together by overlap and sum method, and played out. Figure 1 shows spectrograms of up-gliding noise (left), frequency-steady noise (middle), and down-gliding (right) noise.

The BIAC was always in the temporal center of the noise. For example, to insert a BIAC with a 200-ms duration, the noise section between 900 and 1,100 ms (from the noise onset) in the left-ear channel was substituted by an interaurally independent segment (interaural correlation = 0) with the same parameters. For frequency-gliding noises, the center frequency of the BIAC in up-gliding noise changed from 348 to 459 Hz and the center frequency of the BIAC in down-gliding noise changed from 459 to 347 Hz during the 200-ms BIAC. The center frequency was always 400 Hz, 1,000 ms after the noise onset.

In Experiment 1, the duration of BIAC varied while the overall duration of the noise stimuli was kept at 2,000 ms. The minimum duration required to detect a BIAC (duration threshold) was examined using the frequency-steady noise, up-gliding noise, and down-gliding noise. In Experiment 2, the duration of the BIAC was fixed at 200 ms. When an interaural delay was introduced, a quiet segment with a duration equal to the interaural delay was added to the beginning of the stimulus for the right ear and the end of the stimulus for the left ear. The maximum interaural delay at which the 200-ms BIAC could be detected (delay threshold) was tested for both up-gliding noise and frequency-steady noise in Experiment 2a, and the delay threshold was determined for both down-gliding noise and frequency-steady noise in Experiment 2b.

Sound stimuli were generated using a Creative Sound Blaster PCI128 (Creative SB Audigy 2 ZS, Creative Technology Ltd., Singapore) and delivered by headphones (HD 265 linear, Sennheiser, Germany). The sound intensity was calibrated using a Larson Davis Audiometer Calibration and Electroacoustic Testing System (AUDit and System 824, Larson Davis, Depew, NY, United States). The overall sound level was 63 dB SPL.

### Design and Procedure

The BIAC was perceived as a “central-to-diffuse” change in the noise. The percepts of the BIAC in frequency-gliding were similar to those embedded in frequency-steady noise, except the frequency-gliding noise has a continuous pitch gliding. Note that any auditory event coinciding in time with the BIAC could not be detected when only noise at one ear was delivered. A brief training session was used before Experiment 1 and Experiment 2a and 2b to ensure that each participant understood the instructions and was able to detect the BIAC in each of the three noise types, especially in frequency-gliding noises.

In Experiment 1, the duration threshold for detecting the BIAC was measured for each of the noise types using adaptive two-interval, two-alternative, and forced-choice procedures. In each trial, the BIAC was randomly assigned to one of the two intervals, which were separated by 1,000 ms. The participants’ task was to detect an auditory change in the middle of the noises and identify which of the two intervals contained the change by pressing the left or right button on a response box. The BIAC duration was set to 65 ms at the beginning and manipulated using a three-down one-up procedure: The duration was decreased after three consecutive correct responses and increased after one incorrect response. The initial size of the change in the duration of the BIAC was 16 ms, and the step size was altered by a factor of 0.5 with each reversal in direction of duration change until the minimum value of 1 ms was reached. Feedback was given visually after each trial *via* a LCD monitor in front of the participant. Each adaptive procedure (i.e., a run) was terminated after 10 reversals, and the duration threshold for a run was defined as the arithmetic mean duration across the last six reversals. For each noise type, the arithmetic mean of the duration thresholds for three runs was taken as the duration threshold.

In Experiment 2, the delay threshold for detecting the BIAC was measured using a similar procedure to that for Experiment 1, except that the BIAC duration was fixed at 200 ms. The interaural delay systematically varied in Experiment 2 and was at the beginning set to 0 ms, which is the easiest condition for a listener to detect the BIAC. The interaural delay was increased after three consecutive correct responses and decreased after one incorrect response. The step size started at 16 ms and decreased by half for each reversal until it reached 1 ms. Each adaptive procedure (i.e., a run) was terminated after 10 reversals, and the delay threshold for a run was defined as the arithmetic mean interaural delay across the last six reversals. For each noise type, the arithmetic mean of the delay thresholds for three runs was taken as the delay threshold. A brief training session was also provided before the experiment.

## Results

### Experiment 1

**Figure 2:**
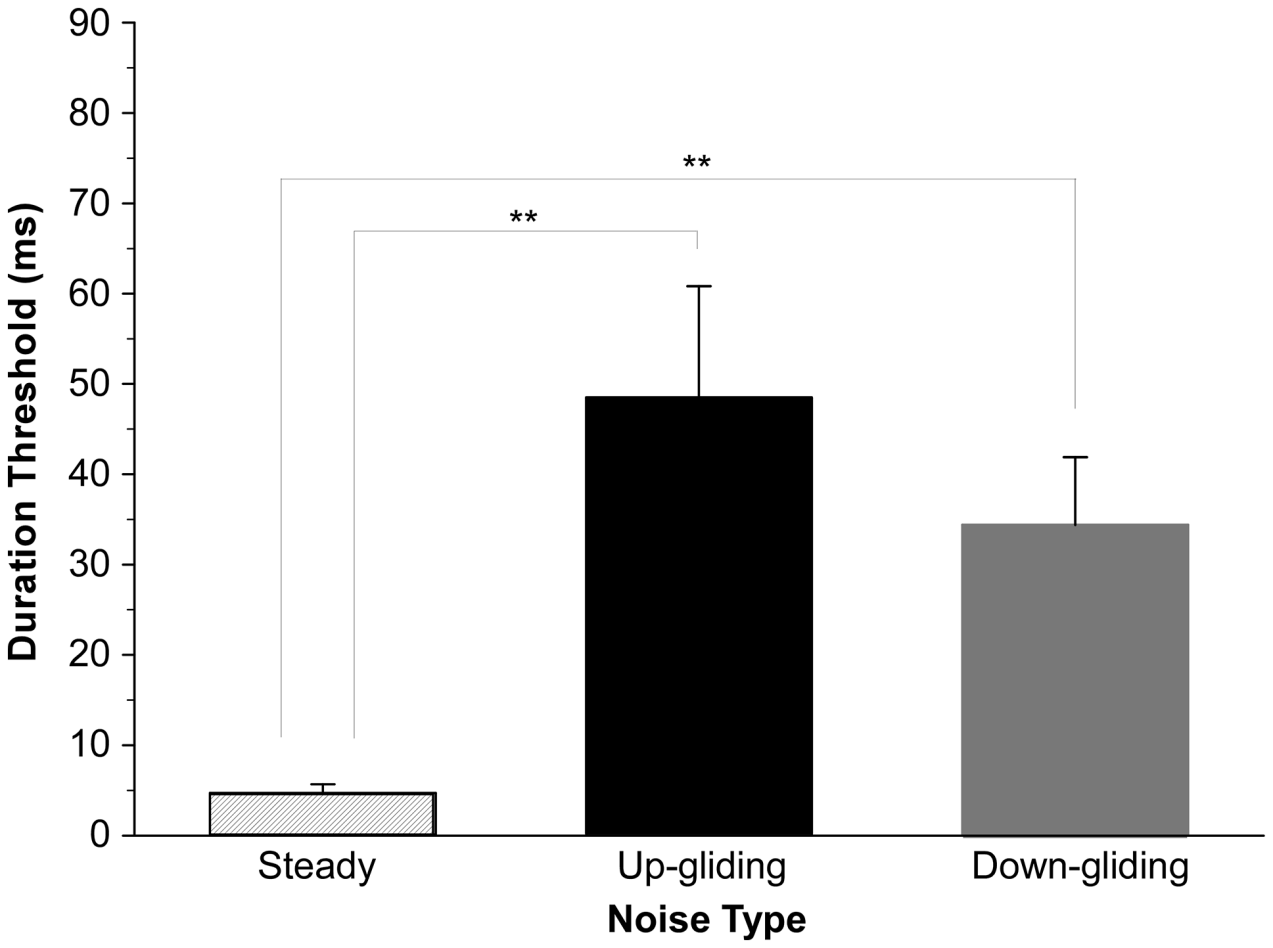
Group-mean duration thresholds for detecting a break in interaural correlation (BIAC) in three types of noises: frequency-steady noise, up-gliding noise, and down-gliding noise (Experiment 1). The error bars represent the standard errors of the means (SEM). ^**^p < 0.01.

The duration thresholds for detecting the BIAC were obtained from 10 participants for each of the noise types when the noise at one ear was delivered simultaneously with that delivered on the other ear. Our results clearly showed that listeners were able to detect a dynamic change in interaural correlation for binaural noises with the unidirectionally varied center frequency. Figure 2 shows the group-mean duration thresholds and standard errors of the mean for each noise type. An important feature was that the binaurally uncorrelated fragments embedded in frequency-gliding noises were much harder to detect than that embedded in frequency-steady noise. The mean thresholds for detecting a BIAC for up-gliding noise, down-gliding noise, and frequency-steady noise were 48.4 ms, 34.4 ms, and 4.7 ms, respectively.

ANOVA across the three conditions of the noise type was performed to determine whether the duration threshold for frequency-gliding noises was much longer than that for frequency-steady noise. The ANOVA showed that the main effect of noise type on the duration threshold was significant [*F* (2,18) = 7.152, *p* < 0.01]. LSD *post-hoc* analyses showed that the duration threshold for detecting the BIAC in frequency-steady noise was significantly shorter than that in up-gliding noise (*p* < 0.01) and down-gliding noise (*p* < 0.01). Moreover, the duration threshold in up-gliding noise was not significantly different from that in down-gliding noise (*p* = 0.354).

### Experiment 2

Among the duration thresholds obtained in Experiment 1, when the interaural delay was zero, the longest duration threshold for up-gliding noise was 119.4 ms and the longest duration threshold for down-gliding noise was 92.9 ms. Thus, it is reasonable to predict that most human listeners are able to detect a 200-ms BIAC in the frequency-gliding noises when the interaural delay is zero. In Experiment 2a, the longest interaural delays at which a 200-ms BIAC could be detected (delay thresholds) were obtained from 21 participants for up-gliding noise and frequency-steady noise. The delay thresholds for down-gliding noise and frequency-steady noise were obtained from another 19 participants in Experiment 2b.

**Figure 3:**
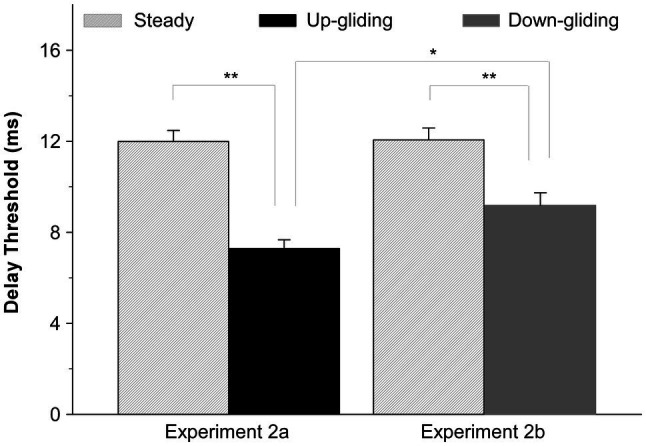
Group-mean interaural delay thresholds for detecting a BIAC in for three types of noises: frequency-steady noise, up-gliding noise, and down-gliding noise (Experiment 2). The error bars represent the SEM. ^*^p < 0.05, ^**^p < 0.01.

In consistent with the results in Experiment 1 that the BIAC in frequency-gliding noises was much harder to detect than that in frequency-steady noise, the maximum interaural delay for detecting the BIAC in frequency-gliding noises was shorter than that for frequency-steady noise. The group-mean delay threshold for detecting the BIAC was 7.3 ms for up-gliding noise and 9.2 ms for down-gliding noise while that for frequency-steady noise was 12.0 ms in Experiment 2a and 12.1 ms in Experiment 2b. Figure 3 shows the group-mean delay thresholds for up-gliding noise and the frequency-steady noise in Experiment 2a, and those for down-gliding noise and frequency-steady noise in Experiment 2b.

A paired t-test was performed to determine if the delay threshold for up-gliding noise was significantly shorter than that for the frequency-steady noise. The paired t-test showed that the difference was significant (*t* = −9.895, *p* < 0.001, Experiment 2a). Similarly, a paired t-test showed that the delay threshold for the down-gliding noise was also significantly shorter than that for frequency-steady noise (*t* = −5.846, *p* < 0.001, Experiment 2b). In contrast to the comparison between the duration threshold for up-gliding noise and that for down-gliding noise, a non-matched samples *t*-test showed that the delay threshold for the up-gliding noise was significantly shorter than that for down-gliding noise (*t* = −2.859, *p* < 0.01).

## Discussion

The primary aim of our study was to determine the sensitivity to a change in interaural correlation when the center frequency of binaural noises varied over time (frequency-gliding noises). The results of this study showed that young adults with normal hearing were able to detect a BIAC embedded in frequency-gliding noises (center frequency: between 100 and 1,600 Hz). However, the duration threshold for frequency-gliding noises was significantly longer than that for frequency-steady noise (center frequency: 400 Hz).

As the detection of a BIAC is determined by the perceptual contrast in the interaural correlation between the uncorrelated segment and the marker (the noise sections flanking the BIAC), the detection difficulty in frequency-gliding noises might be based on the possible decrease in the perceptual contrast between the BIAC and marker in frequency-gliding noises. Given that most models of binaural processing are based on the assumption that sounds are filtered into narrow-band signals and the processing of the binaural information is based on comparisons of interaural differences in a band-by-band manner (Durlach et al., 1986; Stern and Trahiotis, 1995; Akeroyd and Summerfield, 1999), the detection of the BIAC for frequency-gliding noises should be based on both the processing of interaural correlation within the frequency band where the BIAC embedded and the across-band information from the other frequency bands which frequency-gliding noises passed through.

For the processing of interaural correlation within the frequency band where a BIAC embedded (center frequency: 400 Hz), the detection of the BIAC may have been influenced by forward and backward masking from the marker. The duration of the forward fringe for frequency-steady noise would be 997.65 ms, based on the duration of the whole noise, and the mean duration threshold for frequency-steady noise is 4.7 ms. For frequency-gliding noises, however, the effective duration of the marker noise within each frequency band would be affected by the speed of the sweep and the bandwidth of the auditory filters. According to the auditory filter bandwidth of Glasberg and Moore (1990), the bandwidth [equivalent rectangular bandwidth (ERB)], the frequency range of the frequency band (center frequency = 400 Hz), was calculated:

(1) $$ERB\left(f\right)=0.108f+24.7$$

where *f* is the center frequency. Based on the frequency range of the band (366 Hz–434 Hz) and frequency-gliding noises used in this study, the overall duration of frequency-gliding noises in the frequency band (center frequency = 400 Hz) was 125 ms. If the mean duration threshold for frequency-gliding noises is used to estimate the duration of the forward fringe in the frequency band centered on 400 Hz, the duration of the forward fringe is 38 ms for up-gliding noise and 45.3 ms for down-gliding noise. Although no prior study has assessed the effect of forward fringe duration on the detection of a BIAC, the discrimination between binaural noises with different interaural correlation was virtually impossible for durations of 10 and 32 ms (Pollack and Trittipoe, 1959). Although it is possible that the listener can detect the BIAC when only the frequency band where the BIAC embedded was monitored, the detection of BIAC is extremely hard based on the output of binaural processing from the frequency band with the center frequency of 400 Hz according to the findings of Pollack and Trittipoe (1959).

Comparisons of binaural information across frequency play an important role in the binaural lateralization of bandpass noises (Stern and Trahiotis, 1995; Ungan et al., 2019). For example, the interaural time difference of a bandpass noise which is consistent over frequency has been found to be the true interaural time difference of the stimuli (straightness; Stern and Trahiotis, 1995). The detection of the BIAC in frequency-gliding noises probably needs to integrate over a wider frequency range where more binaural information of the marker noises (interaural correlation = 1) is provided than the single frequency band centered on 400 Hz. Around the frequency band with a center frequency of 400 Hz, six frequency bands for the frequency from 82 to 366 Hz and eight frequency bands for the frequency from 434 to 1,724 Hz were included according to the auditory filter bandwidth of Glasberg and Moore (1990). The effective duration of noises in different frequency bands ranged from 78 to 250 ms. It has been shown that the percentage of correct discrimination between binaural noises with an interaural correlation of 0.998 and reference noises with an interaural correlation of 0.922 decreased from 100 to 60% as the noise duration decreased from 316 to 32 ms (Pollack and Trittipoe, 1959). It is speculated that the interaural correlation processing of the marker noises for frequency-gliding noises might be affected by the relatively short duration of marker noises in individual frequency bands.

Our results showed that participants were able to detect a BIAC in frequency-gliding noises even when an interaural delay of several milliseconds was introduced. The human auditory system is able to process binaural cues with interaural delays much longer than those experienced in free-field listening which is usually less than 600 microseconds (Blodgett et al., 1956). Our previous studies have also shown that human listeners can detect a BIAC at larger interaural delays than those experienced in free-field listening for broad-band or narrow-band noises (Huang et al., 2009a,b; Li et al., 2009; Kong et al., 2012, 2015). In the present study, the delay threshold for detecting a fixed-duration BIAC (200 ms) in frequency-gliding noises was significantly shorter than in frequency-steady noise. Fine-structure signals from the leading ear must be maintained (or delayed) for several milliseconds to allow interaural processing of binaural noises with large interaural delays and the maintained information progressively decays as the interaural delay is increased (Huang et al., 2009a; Li et al., 2013). Consistent with the difficulty in detecting a brief BIAC in frequency-gliding noises with no interaural delay, it appears that the maintenance of the fine-structure information for frequency-gliding noises is harder than that for frequency-steady noise.

It has been widely accepted that binaural responses are temporally sluggish when compared with monaural responses (Grantham, 1995; Akeroyd and Summerfield, 1999). The binaural sluggishness is supported by the temporal-window theory that the duration of the binaural temporal window is shown to be significantly longer than that of the monaural temporal window (Akeroyd and Summerfield, 1999). Given the sensitivity to a dynamic change in interaural correlation over time is affected by the binaural temporal window, one possible explanation for the difficulty in detecting the BIAC in frequency-gliding noises might be the increase in the duration of the binaural temporal window for frequency-gliding noises. It has been proved that the processing of interaural correlation makes it harder to perceive the temporal changes in the frequency (Krumbholz et al., 2009). However, whether the binaural temporal window for frequency-gliding noises is broader than that for frequency-steady noise cannot be determined until the just noticeable difference of the interaural correlation for frequency-gliding noises is tested in further studies.

Many sounds in natural environments have frequency modulations, e.g., speech and other communication sounds (Hsieh and Saberi, 2009). Investigation of the interaural correlation processing of sounds with frequency modulations should lead to a better understanding of the mechanism underlying their spatial coding and recognition against a noisy background. For example, considering the detection of a target sound, i.e., speech, against a noisy background when both the target and noise are delivered binaurally through headphones, the detection performance is significantly improved by inverting either the target signal wave or the masking noise wave in one ear (Licklider, 1948). This binaural unmasking effect is suggested to be closely related to the sensitivity to a change in interaural coherence (the degree of similarity of the sound waveforms at the two ears; Durlach et al., 1986). However, the interaural correlation of the stimulus has been found to be a poor predictor of this binaural unmasking effect (van der Heijden and Joris, 2010). Thus, further studies need to be performed to investigate the functional relationship between the interaural correlation of frequency-gliding noises and auditory target detection in noise.

In addition, interaural correlation processing is based on the neural processing of the temporal fine structures (Huang et al., 2009a; Li et al., 2013) which are vulnerable to diseases with auditory neural degeneration, e.g., Alzheimer’s disease (Sinha et al., 1993). Several previous studies have found that the spatial coding and auditory scene analysis were impaired in patients with Alzheimer’s disease (Goll et al., 2012; Golden et al., 2015). The interaural correlation processing of frequency-gliding noises in this study may have clinical significance as a manifestation of the prodromal stage of Alzheimer’s disease.

## Data Availability Statement

The raw data supporting the conclusions of this article will be made available by the authors, without undue reservation.

## Ethics Statement

The studies involving human participants were reviewed and approved by the Committee for Protecting Human and Animal Subjects in the School of Psychological and Cognitive Sciences at Peking University. The patients/participants provided their written informed consent to participate in this study.

## Author Contributions

LF conducted experiments. LK conducted data analysis and manuscript writing. LL contributed to the writing of the manuscript. TQ conducted sound generation and supervised this research project. All authors contributed to the article and approved the submitted version.

### Conflict of Interest

The authors declare that the research was conducted in the absence of any commercial or financial relationships that could be construed as a potential conflict of interest.

## References

[ref1] Aaronson N. L. Hartmann W. M. (2010). Interaural coherence for noise bands: waveforms and envelopes. J. Acoust. Soc. Am. 127, 1367–1372. 10.1121/1.3290991 20329836 PMC2906201

[ref2] Akeroyd M. A. Summerfield A. Q. (1999). A binaural analog of gap detection. J. Acoust. Soc. Am. 105, 2807–2820. 10.1121/1.426897 10335632

[ref3] Blauert J. Lindemann W. (1986). Spatial mapping of intracranial auditory events for various degrees of interaural coherence. J. Acoust. Soc. Am. 79, 806–813. 10.1121/1.393471 3958323

[ref4] Blodgett H. C. Wilbanks W. A. Jeffress L. A. (1956). Effect of large interaural time differences upon the judgment of sidedness. J. Acoust. Soc. Am. 28, 639–643. 10.1121/1.1908430

[ref5] Boehnke S. E. Hall S. E. Marquardt T. (2002). Detection of static and dynamic changes in interaural correlation. J. Acoust. Soc. Am. 112, 1617–1626. 10.1121/1.1504857 12398467

[ref6] Chait M. Poeppel D. Cheveigné A. Simon J. Z. (2005). Human auditory cortical processing of changes in interaural correlation. J. Neurosci. 25, 8518–8527. 10.1523/JNEUROSCI.1266-05.2005 16162933 PMC6725672

[ref7] Culling J. F. Colburn H. S. Spurchise M. (2001). Interaural correlation sensitivity. J. Acoust. Soc. Am. 110, 1020–1029. 10.1121/1.1383296 11519570

[ref8] Durlach N. I. Gabriel K. J. Colburn H. S. Trahiotis C. (1986). Interaural correlation discrimination: II. Relation to binaural unmasking. J. Acoust. Soc. Am. 79, 1548–1557. 10.1121/1.393681 3711454

[ref9] Edmonds B. Culling J. F. (2009). Interaural correlation and the binaural summation of loudness. J. Acoust. Soc. Am. 125, 3865–3870. 10.1121/1.3120412 19507969

[ref10] Gabriel K. J. Colburn H. S. (1981). Interaural correlation discrimination: I. Bandwidth and level dependence. J. Acoust. Soc. Am. 69, 1394–1401. 10.1121/1.385821 7240569

[ref11] Glasberg B. R. Moore B. C. J. (1990). Derivation of auditory filter shapes from notched-noise data. Hear. Res. 47, 103–138. 10.1016/0378-5955(90)90170-t 2228789

[ref12] Golden H. L. Nicholas J. M. Yong K. X. X. Downey L. E. Schott J. M. Mummery C. J.. (2015). Auditory spatial processing in Alzheimer’s disease. Brain 138, 189–202. 10.1093/brain/awu337 25468732 PMC4285196

[ref13] Goll J. C. Kim L. G. Ridgway G. R. Hailstone J. C. Lehmann M. Buckley A. H.. (2012). Impairments of auditory scene analysis in Alzheimer’s disease. Brain 135, 190–200. 10.1093/brain/awr260 22036957 PMC3267978

[ref14] Grantham D. W. (1995). “Spatial hearing and related phenomena,” in Hearing. ed. Moore B. C. J. (London: Academic), 297–345.

[ref15] Hsieh I. H. Saberi K. (2009). Detection of spatial cues in linear and logarithmic frequency-modulated sweeps. Atten. Percept. Psychophysiol. 71, 1876–1889. 10.3758/APP.71.8.1876 19933570

[ref16] Huang Y. Huang Q. Chen X. Wu X. Li L. (2009a). Transient auditory storage of acoustic details is associated with release of speech from informational masking in reverberant conditions. J. Exp. Psychol. Hum. Percept. Perform. 35, 1618–1628. 10.1037/a0015791 19803660

[ref19] Huang Y. Kong L. Fan S. Wu X. Li L. (2008). Both frequency and interaural delay affect event-related potential responses to binaural gap. Neuro Report 19, 1673–1678. 10.1097/WNR.0b013e32831576c7 18806687

[ref18] Huang Y. Lu H. Li L. (2019). Human scalp evoked potentials related to the fusion between a sound source and its simulated reflection. PLoS One 14:e0209173. 10.1371/journal.pone.0209173.g008 30625162 PMC6326413

[ref17] Huang Y. Wu X. Li L. (2009b). Detection of the break in interaural correlation is affected by interaural delay, aging, and center frequency. J. Acoust. Soc. Am. 126, 300–309. 10.1121/1.3147504 19603886

[ref20] Jain M. Gallagher D. T. Koehnke J. Colburn H. S. (1991). Fringed correlation discrimination and binaural detection. J. Acoust. Soc. Am. 90, 1918–1926. 10.1121/1.401671 1960285

[ref21] Jenkins J. J. Strange W. Edman T. R. (1983). Identification of vowels in “vowelless” syllables. Percept. Psychophys. 34, 441–450. 10.3758/bf03203059 6657448

[ref22] Kohlrausch A. Braasch J. Kolossa D. Blauert J. (2013). “An introduction to binaural processing,” in The Technology of Binaural Listening. ed. Blauert J. (Berlin, Heidelberg: Springer), 1–32.

[ref23] Kong L. Xie Z. Lu L. Qu T. Wu X. Yan J.. (2015). Similar impacts of the interaural delay and interaural correlation on binaural gap detection. PLoS One 10:e0126342. 10.1371/journal.pone.0126342 26125970 PMC4488353

[ref24] Kong L. Xie Z. Lu L. Wu X. Li L. (2012). Sensitivity to a break in interaural correlation is co-modulated by intensity level and interaural delay. J. Acoust. Soc. Am. 132, 114–118. 10.1121/1.4734241 22894308

[ref25] Kong Y. Zeng F. (2004). Temporal and spectral cues in mandarin tone recognition. J. Acoust. Soc. Am. 120, 2830–2840. 10.1121/1.2346009 17139741

[ref26] Krumbholz K. Magezi D. A. Moore R. C. Patterson R. D. (2009). Binaural sluggishness precludes temporal pitch processing based on envelope cues in conditions of binaural unmasking. J. Acoust. Soc. Am. 125, 1067–1074. 10.1121/1.3056557 19206881

[ref27] Li L. Huang J. Wu X. H. Qi J. G. Schneider B. A. (2009). The effects of aging and interaural delay on the detection of a break in the interaural correlation between two sounds. Ear Hear. 30, 273–286. 10.1097/AUD.0b013e318198703d 19194287

[ref28] Li H. Kong L. Wu X. Li L. (2013). Primitive auditory memory is correlated with spatial unmasking that is based on direct-reflection integration. PLoS One 8:e63106. 10.1371/journal.pone.0063106 23658664 PMC3639177

[ref29] Licklider J. C. R. (1948). The influence of interaural phase relations upon the masking of speech by white noise. J. Acoust. Soc. Am. 20, 150–159. 10.1121/1.1906358

[ref30] Pollack I. Trittipoe W. J. (1959). Interaural noise correlations: examination of variables. J. Acoust. Soc. Am. 31, 1250–1252. 10.1121/1.1907669

[ref31] Qu T. Cao S. Chen X. Huang Y. Li L. Wu X.. (2013). Aging effects on detection of spectral changes induced by a break in sound correlation. Ear Hear. 34, 280–287. 10.1097/AUD.0b013e31826e4fe1 23132528

[ref32] Sinha U. K. Hollen K. M. Rodriguez R. Miller C. A. (1993). Auditory system degeneration in Alzheimer’s disease. Neurology 43, 779–785. 10.1212/wnl.43.4.779 8469340

[ref33] Stern R. M. Trahiotis C. (1995). “Models of binaural interaction,” in Handbook of Perception and Cognition—Hearing. ed. Moore B. C. J. (San Diego: Academic), 347–386.

[ref34] Ungan P. Yagcioglu S. Ayik E. (2019). Event-related potentials to single-cycle binaural beats of a pure tone, a click train, and a noise. Exp. Brain Res. 237, 2811–2828. 10.1007/s00221-019-05638-4 31451833

[ref35] van der Heijden M. Joris P. X. (2010). Interaural correlation fails to account for detection in a classic binaural task: dynamic ITDs dominate N0Sπ detection. JARO 11, 113–131. 10.1007/s10162-009-0185-8 19760461 PMC2820206

[ref36] Verhey J. L. van de Par S. (2018). Binaural frequency selectivity in humans. Eur. J. Neurosci. 51, 1179–1190. 10.1111/ejn.13837 29359360

[ref37] Wang Q. Lu H. Wu Z. Li L. (2018). Neural representation of interaural correlation in human auditory brainstem: comparisons between temporal-fine structure and envelope. Hear. Res. 365, 165–173. 10.1016/j.heares.2018.05.015 29853322

[ref38] Zeng F. G. Nie K. Stickney G. S. Kong Y. Y. Vongphoe M. Bhargave A.. (2005). Speech recognition with amplitude and frequency modulations. Proc. Natl. Acad. Sci. U. S. A. 102, 2293–2298. 10.1073/pnas.0406460102 15677723 PMC546014

